# P-1733. Demographic and Service-Specific Patterns of Candidemia Species Distribution in Costa Rican Hospitals (2007-2023)

**DOI:** 10.1093/ofid/ofaf695.1904

**Published:** 2026-01-11

**Authors:** Juan Villalobos Vindas, Jose A Castro Cordero, Elvira Segura Retana, Heylin Estrada Murillo, Alvaro A Aviles Montoya, Carlos Ramírez Valverde, Saúl Quirós Cárdenas, Randall G León Solís, Laura Villalobos González

**Affiliations:** Caja Costarricense de Seguro Social, San José, San Jose, Costa Rica; Caja Costarricense de Seguro Social, San José, San Jose, Costa Rica; Caja Costarricense de Seguro Social, San José, San Jose, Costa Rica; Caja Costarricense del Seguro Social, La Unión, Cartago, Costa Rica; Caja Costarricense de Seguro Social, San José, San Jose, Costa Rica; Caja Costarricense del Seguro Social, La Unión, Cartago, Costa Rica; CCSS, San Jose, San Jose, Costa Rica; Caja Costarricense de Seguro Social, San José, San Jose, Costa Rica; Caja Costarricense de Seguro Social, San José, San Jose, Costa Rica

## Abstract

**Background:**

Understanding the demographic and service-specific distribution of candidemia is essential for targeted prevention strategies. This study characterizes candidemia patterns across different patient populations and hospital settings in Costa Rica.

**Figure:**
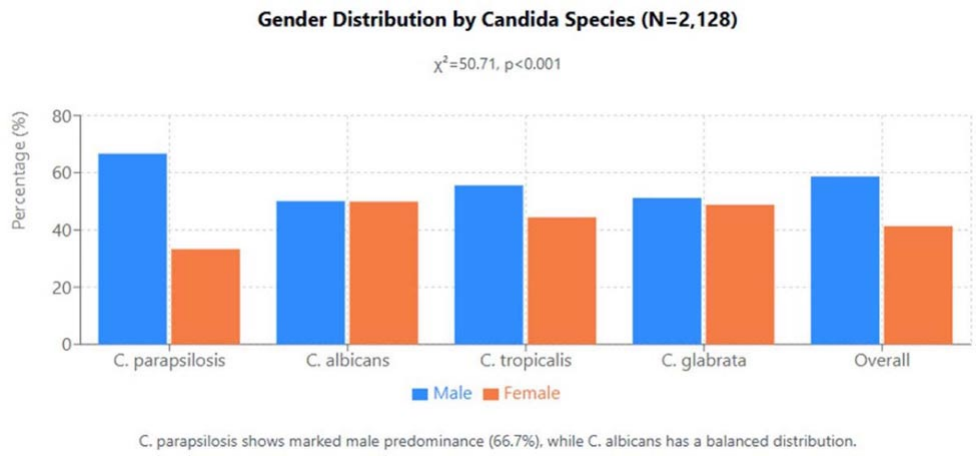
Gender Distribution by Candida Species (N=2,128) Gender Distribution by Candida Species (N=2,128)

**Figure:**
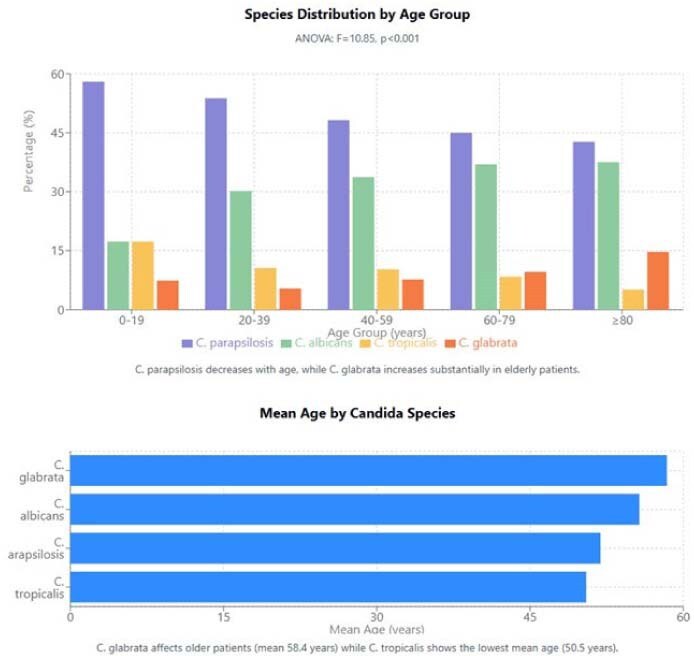
Species Distribution by Age Group Species Distribution by Age Group

**Methods:**

We analyzed 2,128 candidemia cases from two tertiary hospitals, examining species distribution by sex, age, hospital service, and clinical outcomes.

**Figure:**
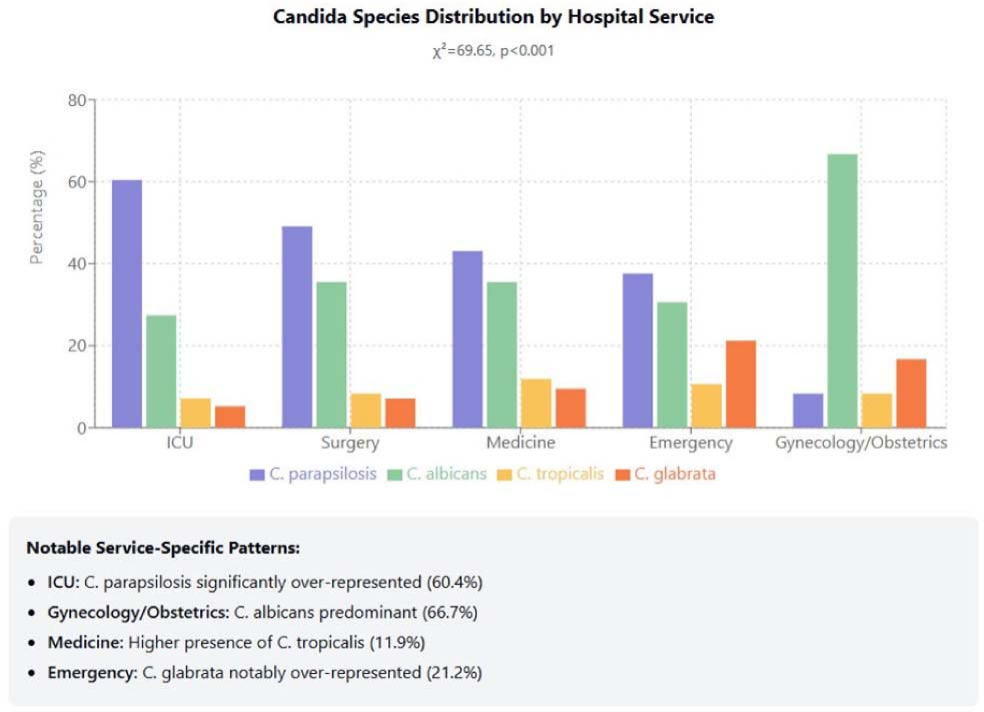
Candida Species Distribution by Hospital Service Candida Species Distribution by Hospital Service

**Results:**

Significant gender differences were observed in species distribution (χ²=50.71, p< 0.001): males represented 58.7% of all cases, with C. parapsilosis showing marked male predominance (66.7%) compared to the balanced distribution of C. albicans (50.1% male). Age-specific patterns revealed significant differences between species (ANOVA: F=10.85, p< 0.001): C. glabrata affected older patients (mean 58.4 years) while C. tropicalis showed the lowest mean age (50.5 years). The proportion of C. parapsilosis decreased with age (58.0% in 0-19 years vs. 42.7% in ≥80 years), while C. glabrata increased substantially (7.4% to 14.7%). Service-specific distribution showed distinctive patterns (χ²=69.65, p< 0.001): C. parapsilosis was significantly over-represented in ICUs (60.4%), C. albicans dominated in Gynecology/Obstetrics (66.7%), C. tropicalis showed higher presence in Medicine services (47.1%), and C. glabrata was notably over-represented in Emergency departments (21.2%). Inter-hospital differences were significant (χ²=95.56, p< 0.001), with C. parapsilosis predominating at Hospital México (52.3%) versus C. albicans at Hospital San Juan de Dios (38.6%).

**Conclusion:**

This study reveals distinct epidemiological profiles of Candida species across different demographic groups and hospital services. These patterns suggest the influence of specific risk factors in different patient populations and clinical settings, highlighting the need for tailored prevention strategies and empirical treatment approaches based on patient characteristics and hospital environment.

**Disclosures:**

All Authors: No reported disclosures

